# Utilizing Latent Class Analysis to Assess the Association of Intersectional Stigma on Mental Health Outcomes Among Young Adult Black, Indigenous, and Sexual Minority Women of Color

**DOI:** 10.1089/lgbt.2022.0083

**Published:** 2023-08-30

**Authors:** Casey D. Xavier Hall, Rachel Harris, Paul Burns, Candace Girod, Kathryn M. Yount, Frankie Y. Wong

**Affiliations:** ^1^ Center of Population Sciences for Health Equity, College of Nursing, Florida State University, Tallahassee, Florida, USA.; ^2^ Institute for Sexual and Gender Minority Health and Wellbeing, Northwestern University, Chicago, Illinois, USA.; ^3^ Department of Medical Social Sciences, Feinberg School of Medicine, Northwestern University, Chicago, Illinois, USA.; ^4^ College of Social Work, Florida State University, Tallahassee, Florida, USA.; ^5^ John D. Bower School of Population Health, Department of Population Health Science, University of Mississippi Medical Center, Jackson, Mississippi, USA.; ^6^ Independent Researcher, Atlanta, Georgia, USA.; ^7^ Hubert Department of Global Health, Rollins School of Public Health, Emory University, Atlanta, Georgia, USA.; ^8^ Department of Sociology, Emory University, Atlanta, Georgia, USA.; ^9^ Department of Psychology, University of Hawai'i at Mānoa, Honolulu, Hawaii, USA.; ^10^ Department of Epidemiology, Fudan University, Shanghai, China.

**Keywords:** depression, intersectional stigma, intersectionality, sexual minority women, stress

## Abstract

**Purpose:**

Discrimination has detrimental effects on mental health, particularly among Black, Indigenous, and people of color who are also sexual minority women (BIPOC SMW); however, measurement of multiple intersecting forms of discrimination (e.g., race, gender, and sexual identity discrimination among BIPOC SMW) poses methodological challenges. This analysis uses latent class analysis (LCA) to examine the influences of discrimination on mental health in a convenience sample of BIPOC SMW.

**Methods:**

Online survey data from BIPOC SMW aged 18–29 years (*n* = 324) were used to estimate latent classes for discrimination type (race, gender, and sexual identity). Data for this study were collected from July to October 2018. Adjusted linear regressions examined the influences of discrimination profiles on perceived stress and depressive symptoms.

**Results:**

Utilizing LCA, the following four classes emerged: (1) low discrimination; (2) mid-level discrimination; (3) high racial, medium gender, and low sexual identity discrimination; (4) high discrimination. Classes 3 and 4 were positively associated with perceived stress and depressive symptoms relative to Class 1 in adjusted models.

**Conclusion:**

This analysis highlights the importance of intersectionality and the adverse impact of multiple forms of discrimination on mental health outcomes for BIPOC SMW. Respondents reporting higher levels of racial or multiple forms of discrimination had poorer mental health outcomes. LCA is a promising analytical tool for investigating intersectional stigma and discrimination. There is an urgent need to develop tailored, culturally appropriate intersectional mental health interventions to address the multiple identities and oppressions faced by BIPOC SMW.

## Introduction

Black, Indigenous, and/or people of color who are also sexual minority women (BIPOC SMW) have multiple identities that hold unique positions, as defined by society.^1,2^ These positions may impact their experiences such as stigma^1^ and power in social interactions.^2–6^ BIPOC SMW do not simply exist as any one identity (e.g., sex, race, or sexual orientation) nor is their experience of mutually influential identities easily disentangled. Thus, it is insufficient to understand their experiences through a single identity.^2^ Research has generated evidence supporting a link between intersectional stigma and mental health inequities.^7,8^ Understanding the intersectional stigma experiences of BIPOC SMW is important, because such experiences may contribute to elevated rates of mental health concerns among BIPOC SMW.^9^ To date, however, relatively few studies have characterized the etiological links between multiple forms of stigma and mental health with a focus on BIPOC SMW, and even fewer studies have done so using intersectional analytical methods.^5,6,10–12^

### BIPOC SMW and discrimination

Intersectional stigma refers to the co-occurrence or convergence of multiple dimensions of discrimination (e.g., sexism, racism, heterosexism) or discrimination that can be attributed to intersecting marginalized social positions (e.g., Black lesbian woman).^1,4,13^ It is well known that sexual minority (SM) persons are exposed to heterosexist discrimination;^14,15^ however, BIPOC SMW encounter gender- and race-based discrimination in addition to heterosexist discrimination.^1,7,8^ Notably, SMW are impacted by sexism, a component of intersectional stigma that is not often captured in research centering SM men or broader SM populations despite its documented impact on SMW's mental health.^16^

Research needs to better document how sexism contributes to experiences of intersectional discrimination (such as gendered racism^17^) or how SMW experiences likely vary from experiences from heterosexual women counterparts.^10,18^ Studies have found that BIPOC SMW report a higher frequency of discrimination experiences across multiple dimensions (i.e., sexism, racism, heterosexism) when compared with White SMW and BIPOC SM men.^8^ In addition, relative to their White counterparts, research suggests that BIPOC SMW are more likely to experience any discrimination,^7^ multiple dimensions of discrimination, and a wider scope of discriminatory encounters (e.g., in more settings and circumstances).^7,8,14^ Overall, the literature indicates that intersecting systems of marginalization may function to expose BIPOC SMW to a disproportionate amount of discrimination relative to other SM subgroups.^5,19^

### BIPOC SMW and mental health

Mental health is a serious concern among BIPOC SMW. Young SMW experience higher rates of depressive symptoms, anxiety symptoms, and suicidality relative to heterosexual peers.^20,21^ As much as 48% of Black SMW and 60% of Latina SMW met the diagnostic criteria for depression in a community sample.^9^ Moreover, BIPOC SMW face many barriers to accessing mental health services, including discouraging cultural norms, intersectional stigma enacted by providers, and structural disadvantage.^22^ Given the prevalence of depression among BIPOC SMW and lack of access to culturally appropriate care, it is critical to better understand the influence of intersectional factors on mental health outcomes of BIPOC SMW.

Research on SMW suggests that heterosexism, sexism, and racism independently contribute to various mental health outcomes, including depressive symptoms and stress.^7,18,23–28^ Moreover, study findings indicate that experiencing intersectional stigma may further increase vulnerability to poor mental health outcomes above and beyond a single dimension.^10,29^ For example, greater exposure to discrimination, totaled across multiple dimensions, has been linked to lower psychological well-being, depression, chronic strain, and greater stressful life events.^8,29^

Furthermore, studies have found that attributing experienced discrimination to more than one identity (e.g., sexual orientation, race, gender) is predictive of worse mental health outcomes.^7,8^ However, findings are complex. Although BIPOC SMW are more likely to experience both a higher frequency of discrimination and multiple dimensions of discrimination, overwhelmingly, studies have found no differences^30–33^ and at times better mental health outcomes among BIPOC SMW relative to White counterparts^9,30,34^ These seemingly paradoxical findings in a yet limited literature highlight the need to better understand intersectional stigma and mental health outcomes for BIPOC SMW.

### Intersectional stigma and methodological challenges

Intersectionality scholars have long argued that to empirically explain the effect of intersectional stigma on outcomes, differing yet mutually constitutive dimensions of discrimination must be accounted for in a holistic way.^5,6,12^ To date, empirical investigations on the effects of intersectional stigma have often relied on analytical approaches that have been critiqued in relation to the intersectional framework, namely additive and multiplicative models.^10,12,35^ The exclusive use of these approaches has been critiqued based on their perceived incompatibility with intersectional frameworks, which challenges disentanglement of discrimination experiences at the intersection of identities.^2,4,6,10,12^ In relation to BIPOC SMW, the use of non-intersectional analytical models may be insufficient in capturing the complexity of intersectional stigma, thus inaccurately capturing experiences of discrimination at the intersection of identities and their influences on mental health.^4,6,36^ Intersectional theorists and methodologists continue to grapple with various approaches to measuring and analyzing intersectional stigma.^2,35^

### The current study

Latent class analysis (LCA) has emerged as a promising analytical tool for investigating intersectional stigma and discrimination.^4,6^ LCA is a person-centered analytical technique, useful for examining complex social phenomena, such as the effects of intersectional stigma.^4,6,37,38^ Researchers have utilized LCA to identify discrimination profiles among samples of multiply marginalized individuals and examine associations between discrimination profiles and various outcomes;^4,6,37^ however, to the best of our knowledge, no study has used LCA to examine intersectional stigma in a sample of BIPOC SMW.

Given that few studies have investigated the effect of intersectional stigma on the mental health of BIPOC SMW,^10^ and even fewer have done so using analytical methods consistent with intersectional perspectives,^6^ the purpose of the current study was to utilize LCA to (a) identify distinct discrimination profiles among a racially and ethnically diverse sample of young SMW, based on their reported experiences of enacted discrimination, and (b) examine associations between discrimination profiles and two mental health related outcomes: (1) perceived stress and (2) depressive symptoms.

## Methods

### Procedures

In this secondary data analysis, we utilized survey data from an existing study^39^ conducted with a racially and ethnically diverse sample of BIPOC SMW. Participants were recruited in July–October 2018 through paid advertisements on three social media platforms and unpaid online advertisements in various public forums. Advertisements described the study as a “women's health study” and provided a link to a landing page where participants screened for eligibility and provided informed consent electronically. Anyone who clicked on the advertisement was able to enter a raffle for one of nine $25 gift cards. The self-administered electronic survey took an average of 11 minutes to complete. Eligibility requirements imposed by the parent study included: (1) English-speaking, (2) identifying as a woman, (3) residence in the U.S. Southern Census Region, and (4) being between the ages of 18–29 years. Additional inclusion criteria for this analytic sample included: (1) identifying as BIPOC (Black, Hispanic/Latina, Asian/Pacific Islander, or Multi-racial). The Emory University Institutional Review Board approved this study and provided ethical oversight.

### Sample

Of the 1403 people who consented to participate, 324 (23.0%) met the inclusion criteria for the current analyses. Sample characteristics are presented in Table 1.

**Table 1.** Demographics of Young Adult Black, Indigenous, and Sexual Minority Women of Color (*n* = 324)

Variable	*n *(%)	Mean (SD)
Age, years		22.3 (3.2)
Gender modality		
Transgender woman	4 (1.2)	
Cisgender woman	320 (98.8)	
Race		
Hispanic/Latina	143 (44.1)	
Black	101 (31.2)	
Multiple races	56 (17.3)	
Other racial identity (Asian or Pacific Islander, American Indian or Alaskan Native)	24 (7.4)	
Sexual identity		
Bisexual	135 (41.7)	
Pansexual	80 (25.0)	
Gay/Lesbian	54 (16.7)	
Queer	39 (12.0)	
Other sexual identity	14 (4.3)	
Education		
Graduate degree	87 (26.9)	
Bachelor's degree	153 (47.2)	
Associates or some college	66 (20.4)	
High school or less	18 (5.6)	
Racial discrimination (mean score)		1.7 (1.4)
Sexual identity discrimination (mean score)		0.9 (0.9)
Gender discrimination (mean score)		1.5 (1.6)
Stress score (sum score)		25.4 (5.9)
Depression score (sum score)		17.9 (7.2)

SD, standard deviation.

### Measures

#### Discrimination

The 9-item Everyday Discrimination Scale was used to measure experiences of discrimination.^40^ The scale was modified to ask about discrimination relative to specific identities by adding a phrase such as “due to your race or ethnicity” to each prompt. Use of the modified scale has been published previously in a number of studies.^39,41^ The full scale was repeated for three identities—race/ethnicity, gender, and sexual identity. The scale assesses experiences of discrimination, such as “You are treated with less respect than other people due to your sexual orientation” during the preceding year. The six response options ranged from “never” (0) to “almost every day” (5). Scores on the summative scale ranged from 0 to 45, with higher scores reflecting more frequent experiences of discrimination. The Cronbach's alpha in the current sample was 0.96 for race, 0.91 for gender, and 0.93 for sexual identity.

#### Stress

We utilized the Perceived Stress Scale.^42^ The 10-item scale measures symptoms of stress, such as “felt nervous or stressed,” with a 5-level Likert response scale, ranging from “Never” to “Very Often,” for the past year.^42^ There were no missing data. The Cronbach's alpha in the sample was 0.85.

#### Depressive symptoms

Depressive symptoms were measured with the short form of the Center for Epidemiological Studies Depression Scale, which has 12 items^43^ assessing depressive symptoms such as “I could not get going” during the preceding week. The four response options range from “rarely or none of the time” (0) to “most or almost all of the time” (3). Responses are summed across items and scores range from 0 to 36. A score of 0 to 11 represents minimal symptoms, 12 to 20 represents elevated symptoms, and 21 to 36 represents very elevated symptoms.^43^ The Cronbach's alpha in the current sample was 0.86.

#### Demographics

Age was measured in years (18–29 years). Self-identified race was measured as non-Hispanic Black, Latino or Hispanic, American Indian or Alaskan Native, Asian or Pacific Islander, and biracial/multiracial. For analyses, American Indian or Alaskan Native, Asian/Pacific Islander, and people who selected “other racial identity” were combined into one category due to low numbers. Education was measured as high school diploma or less, some college or an associate degree, or a bachelor's degree or a graduate degree. All women who are included in the analytic sample indicated that they identify as SMW. Participants were asked to indicate at least one sexual identity. Sexual identity was then categorized as (1) Bisexual, (2) Pansexual, (3) Queer, (4) Gay/Lesbian, and (5) individuals who indicated multiple identities or less frequently indicated identities (e.g., demisexual). The fifth group was categorized as “other sexual identity” due to small numbers. Participants indicated their gender modality which in this sample included cisgender and transgender women.

### Analyses

Univariate and bivariate analyses were used to examine the distributions of the variables included in this analysis and the assumptions for statistical tests. These procedures were conducted in SAS version 9.4. First, the latent classes were estimated with maximum likelihood estimation and bootstrapping methods in MPlus 8. Items used to estimate latent classes included the three discrimination scales. Decisions about class number selection were guided by the following seven criteria: (1) all class membership sizes being above 20 participants or roughly 5% of the sample, (2) entropy of 0.80 or higher, (3) Vuong–Lo–Mendell–Rubin likelihood ratio test, (5) Lo–Mendell–Rubin test, (6) parametric bootstrapped likelihood ratio test, and, importantly, (7) conceptual interpretability of latent classes.^44^ Resulting latent classes were then exported as a dependent variable and used in regressions for perceived stress and depressive symptom outcomes, controlling for demographic characteristics.

## Results

### Univariates

The mean scale scores are reported in Table 1. The mean stress score was 25.4 indicating an average score in the moderate range (14–26).^45^ The mean depression score was 17.9 indicating elevated depressive symptoms.^43^

### Latent class estimation

The latent class estimation resulted in a four-class model with a Bayesian information criterion of 2813.8 and entropy of 0.84. Indices are presented in Table 2. All three fit indices were significant, including the Vuong–Lo–Mendell–Rubin likelihood test (241.2, *p* < 0.001), the Lo–Mendell–Rubin test (48.1, *p* = 0.05), and the bootstrapped likelihood ratio test (50.2, *p* < 0.001). One resulting class in the final model was lower than *n* = 20; however, in alignment with current best practices, we selected the four-class model given the strong fit indices and conceptual fit of the resulting models.^46^

**Table 2.** Fit Statistics for Possible Latent Class Solutions by Number of Classes

Class	BIC	Adjusted BIC	LMR-LRT		BLRT		Entropy
			Estimate	*p*	Estimate	*p*	
1	—	—	—	—	—	—	—
2	2885.047	2853.328	196.979	0.0005	205.498	0.0000	0.814
3	2842.599	2798.192	62.853	0.3465	65.572	0.0000	0.856
**4**	**2813.836**	**2756.742**	**49.735**	**0.0105**	**51.885**	**0.0000**	**0.838**
5	2813.007	2743.225	22.959	0.8504	23.952	0.0000	0.827
6	2795.848	2713.379	38.612	0.0371	40.281	0.0000	0.846
7	2795.669	2700.512	22.336	0.4818	23.302	0.0000	0.848

Lower BIC and adjusted BIC values indicate a better fitting model. Significant LMR and BLRT indicate a preference for the current model over the model with one less class. Bold text is the selected solution.

BIC, Bayesian information criterion; BLRT, bootstrapped likelihood ratio test; LMR-LRT, Lo-Mendell-Rubin likelihood ratio test.

**FIG. 1.**
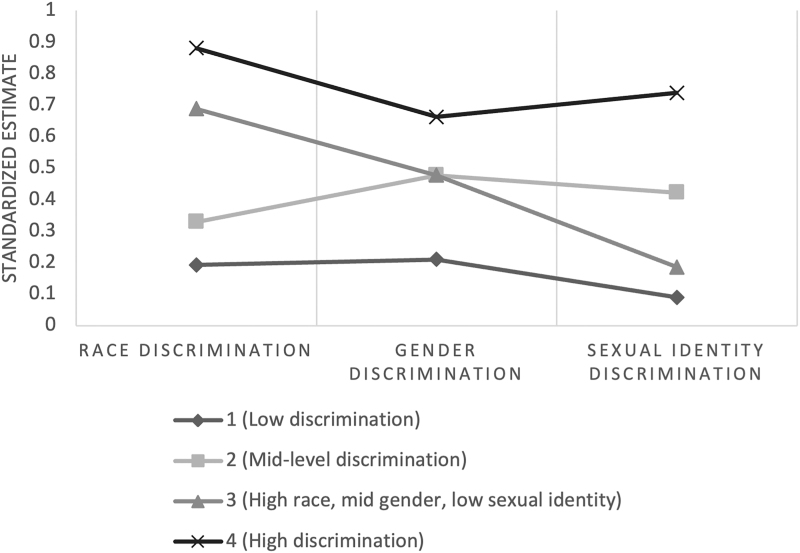
Four-class solution with standardized estimates by discrimination type.

The four resulting classes included (1) low discrimination; (2) mid-level discrimination; (3) high racial, mid-level gender, low sexual identity discrimination; and (4) high discrimination (Fig. 1). Class 1 was the largest (*n* = 203, 62.7%), followed by Class 3 (*n* = 58, 17.9%), Class 2 (*n* = 50, 15.4%), and Class 4 (*n* = 13, 4.0%).

### Stress

In the unadjusted model, latent classes were significantly associated with perceived stress such that Class 3 (β = 3.1, standard error [SE] = 0.8, *p* < 0.01) and Class 4 (β = 7.8, SE = 1.6, *p* < 0.01) had higher stress scores than Class 1 (Table 3). After adjusting, latent classes remained significantly associated with stress such that Class 3 (β = 2.8, SE = 1.1, *p* < 0.01) and Class 4 (β = 7.7, SE = 2.0, *p* < 0.01) had higher stress scores than Class 1.

**Table 3.** Regressions for Perceived Stress and Depressive Symptoms Among Young Adult Black, Indigenous, and Sexual Minority Women of Color (*n* = 324)

Variable	Perceived stress						Depressive symptoms					
	Bivariate			Multivariable*^a^*			Bivariate			Multivariable*^a^*		
	β	SE	*p*	β	SE	*p*	β	SE	*p*	β	SE	*p*
Latent class (based on discrimination scales)												
1 (Low discrimination)	Ref.			Ref.			Ref.			Ref.		
2 (Mid-level discrimination)	1.0	0.9	0.24	1.2	1.1	0.17	2.4	1.1	0.03	2.5	1.1	0.19
3 (High race, mid gender, low sexual identity)	3.1	0.8	0.0003	2.8	1.1	0.001	3.9	1.0	0.001	3.6	1.0	0.001
4 (High discrimination)	7.8	1.6	<0.0001	7.7	2.0	<0.0001	9.8	2.0	<0.0001	9.7	2.0	<0.0001
*R* ^2^				0.14						0.14		

^a^ Adjusting for age, racial identity, gender, and sexual identity.

SE, standard error.

### Depressive symptoms

In the unadjusted model, latent classes were significantly associated with depressive symptoms such that Class 2 (β = 2.4, SE = 1.1, *p* = 0.03), Class 3 (β = 3.9, SE = 1.0, *p* < 0.01), and Class 4 (β = 9.8, SE = 2.0, *p* < 0.01) had higher depression scores than Class 1. In the adjusted model, latent classes were significantly associated with depressive symptoms such that Class 3 (β = 3.6, SE = 1.1, *p* < 0.01) and Class 4 (β = 9.7, SE = 2.0, *p* < 0.01) had higher depression scores than Class 1.

## Discussion

This analysis is one of the first published studies to employ an LCA approach to measuring intersecting forms of discrimination among BIPOC SMW by estimating latent classes based on gender, racial, and sexual identity discrimination. These results suggest by using a participant-centered analytic approach (e.g., LCA), we can observe more nuanced qualitative differences in discrimination profiles among BIPOC SMW and their differential impact on health outcomes.

Our findings align with the literature suggesting that higher levels of discrimination are associated with negative health outcomes among BIPOC SMW.^1,7,8,14,47^ However, LCA may provide a more nuanced understanding suggesting that classes with higher levels of discrimination in at least one category (Classes 3 and 4) had significantly higher negative mental health indicators, with those (Class 4) having the highest levels of discrimination across categories with the worst mental health outcomes. Moreover, in post hoc analyses with Class 3 as the referent category, we observed significantly higher stress and depression scores among participants in Class 4 relative to Class 3.

This finding suggests that not only high discrimination, but high discrimination across multiple discrimination targets (race, gender, and sexual identity) has a detrimental impact on mental health in BIPOC SMW. Although race, gender, and sexual identity discrimination have been found to independently affect the health of BIPOC SMW,^48^ not all three forms are consistently captured in intersectional research. For example, studies in which BIPOC SMW are included in broader samples or are characterized by a single identity such as SM status may not fully capture the impact of sexism or racism among BIPOC SMW. This analysis underlines the importance of considering the combined influence of race, gender, and sexual identity discrimination in the lives of BIPOC SMW.

This analysis is informative to approaches of addressing intersectional stigma in that LCA allows for parsing of identity-based attributions and frequency of attribution while also establishing qualitatively different latent classes based on discrimination profiles to examine the combined influences on health. Some have suggested that researchers should move toward intersectional measurement where the intersection of identity is viewed holistically (e.g., measuring discrimination uniquely targeting the combination of Black, queer, women identities as opposed to measuring discrimination targeting each specific identity and summing these measures).^2,4,47^ Although an LCA approach does not fully address this critique, it provides another tool in addition to existing additive and intersectional approaches in an ever-expanding toolbox.^2^

This analysis emphasizes that BIPOC SMW do not simply exist as a single identity (e.g., sex, race, or sexual orientation), but rather at the intersection of multiple marginalized identities, the marginalization of which contributes to various mental health outcomes, including depressive symptoms and stress.^7,18,23–28^ As BIPOC SMW are disproportionately impacted by depression, anxiety, and suicidality, it is critical to understand and address the factors contributing to this disparity.^20–22^ Moreover, BIPOC SMW reside in higher concentrations in states lacking legal protections, for example, the South has the largest concentration of states without legal protections against discrimination of SM populations.^49^

Interventions that seek to address a single type of discrimination may not be effective at diminishing the mental health impact of discrimination among BIPOC SMW.^4,50^ Recent literature reviews on racial and sexual identity stigma emphasize social support interventions as beneficial buffers from the mental health effects of discrimination;^51,52^ however, given the nature of intersectional forms of discrimination, interventions need to be adapted to incorporate the perspectives and needs of BIPOC SMW.^50^

### Limitations

The findings of this study should be considered in light of several limitations. First, our data were cross-sectional, precluding causal interpretations. Second, the final latent classes had strong fit indices across the board; however, Class 4 had a limited size (*n* = 13). The general recommendation is to consider class size in balance with other criteria, including conceptual coherence.^46^ Future studies should seek to replicate the LCA in larger samples. Third, this sample was age and geographically restricted by the parent study inclusion criteria, which thus limited the generalizability of these findings to the geographies, age ranges, and calendar period for the present analysis.

Given that experiences of discrimination and outcomes related to perceived stress and depressive symptoms may vary over the life course, replicating this study in other age groups of BIPOC SMW and in longitudinal cohort studies will be important to deepen our understanding of how historical, socio-contextual, and age-related experiences of discrimination relate to mental health in BIPOC SMW. Fourth, a strength of LCA methods is the ability to consider the combined effect of multiple forms of discrimination; however, in the present analysis, we were unable to fully account for differences by race/ethnicity or sexual identity due to sample restrictions or specific types of overlap in discrimination (e.g., gendered racism) due to the way discrimination is measured. Future studies may consider examining differences in latent classes across identities and overlapping forms of discrimination. Finally, the small size of the subsample of transgender women prevented us from drawing conclusions specific to transgender women.

## Conclusion

BIPOC SMW experience intersectional stigma, including but not limited to gender, sexual identity, and racial discrimination. The present analysis suggests that BIPOC SMW who have discrimination profiles characterized by elevated discrimination across multiple types may experience a greater degree of stress and depressive symptoms relative to those who report lower rates of discrimination. Multilevel intersectional interventions are urgently needed to address the intersectional stigma faced by BIPOC SMW to improve their mental health and well-being.
